# Climatic and edaphic controls over soil δ^15^N in temperate grassland of northern China: A PLS-PATH analysis

**DOI:** 10.1371/journal.pone.0265795

**Published:** 2022-10-31

**Authors:** Xianzhao Liu, Zhengying Luo, Tianhao Wang, Qing Su

**Affiliations:** 1 School of Earth Science and space information Engineering, Hunan University of Science and Technology, Xiangtan, Hunan, China; 2 School of Life and Health Science, Hunan University of Science and Technology, Xiangtan, Hunan, China; Jinan University, CHINA

## Abstract

Identifying the impact path of climate and soil factors on soil δ^15^N is very crucial for better understanding the N turnover in soils and the integrated information about ecosystem N cycling. Many studies have showed that climate and soil variables influence the change of soil δ^15^N. However, most of the existing studies focused on the overall impact of factor on soil δ^15^N, without distinguishing between the direct and indirect effect. Although scholars have studied the relationships among temperature, precipitation, soil N, soil pH, and soil δ^15^N rather than estimating all the causal relationships simultaneously. To answer the above-mentioned questions, a regional-scale soil collection was conducted across a temperate grassland in northern China. Meanwhile, a PLS-PATH analysis was utilized to evaluate the direct and indirect effects of various factors on soil δ^15^N and to explore the causal relationships among variables. The results showed that along the transect, mean annual precipitation (MAP) and mean annual temperature (MAT) directly and significantly reduced soil δ^15^N, and indirectly affected soil δ^15^N through their effects on soil pH, soil clay, soil N and soil C/N. Soil C/N ratio has a significant direct impact on soil δ^15^N with a negative correlation. Soil clay, soil N content, and soil pH have a total positive effect on soil δ^15^N, but the total positive impact of soil pH is very weak because it has a negative indirect impact on soil δ^15^N by affecting soil clay, soil N and soil C/N ratio. The total influence is, in order, MAP > MAT > soil C/N > soil clay > soil N > soil pH (in absolute value). The above results will provide valuable information about ecosystem N cycle in temperate grassland of northern China.

## Introduction

With the rapid development of isotope measurement technology, the analysis of soil nitrogen isotope (expressed as soil δ^15^N) has become an effective tool to explore the nitrogen (N) dynamics and soil development. Soil δ^15^N can potentially provide valuable information about N cycle in soil ecosystem, because it is mainly based on overall isotopic fractionation during microbial degradation and transformation or the preferential decomposition of the substrates depleted in ^15^N [1–8]. In general, compared with the substrate with lower decomposition, the older soil with higher microbial treatment is enriched in ^15^N [9,10]. Since ^14^N is preferentially lost from the ecosystem, resulting in the enrichment of soil ^15^N in the ecosystem with high N cycle openness, soil δ^15^N can be used as a valid indicator of ecosystem N cycle openness [11]. Generally, the N cycle in arid areas is more open than that in humid areas because soil δ^15^N value is often negatively correlated with precipitation on different spatial scales. Additionally, without considering the inputs of anthropogenic N and the influence of plant residues, the variation in soil δ^15^N in natural ecosystems is largely controlled by climate and soil factors during microbial decomposition and nitrogen turnover [12–14]. Therefore, the signature of ^15^N in soil integrates a variety of information about the ecosystem N cycling [15,16]. Unfortunately, the influential mechanism that drive the relationships between climatic and edaphic variables and soil δ^15^N values in terrestrial ecosystem has not yet been fully understood, which affects the interpretation of global or regional patterns of N-cycling.

To understand the driving factors controlling soil δ^15^N along natural environment gradients, numerous scholars have conducted extensive research at regional and global scales [13,17–22]. Most results have revealed that climatic and edaphic factors, including mean annual temperature (MAT), mean annual precipitation (MAP), soil pH, soil C and N content and soil texture, can affect soil δ^15^N values by affecting N transformation and release [13,23,24]. For instance, on a global scale, climatic variables can control soil δ^15^N, with values increasing in response to decreasing MAP and increasing MAT, which will enhance the process leading to N loss of but discriminate against ^15^N loss [12,21,25]. Recent studies indicated that climate factors control about 50% of the variation of soil δ^15^N across temperate grassland in northern China. It was found that soil δ^15^N decreased with the increase of MAP and MAT [26]. Further studies exhibited that drought can nonlinearly shape soil δ^15^N in arid and semi-arid grassland [11,22]. Therefore, on a certain spatial scale, soil δ^15^N values across climate gradient can mirror the relationship between N losses and turnover [23]. Besides climatic factors, soil factors have been demonstrated to affect the pattern of soil δ^15^N [12]. For example, soil δ^15^N values are negatively related to soil organic carbon and positively associated with soil N contents [10,13,27,28]. Soil pH may affect soil N availability via its influences on microbial nitrification, denitrification and anaerobic ammonium oxidation in terrestrial ecosystems, thus controlling the δ^15^N values in soils [9,24,25,29]. Similarly, soil texture can also impact soil N turnover by its indirect impact on other soil physicochemical properties (e.g., soil water, oxygen concentration and soil N content), thus driving changes in isotope values Similarly, soil texture can also indirectly affect other soil physical and chemical properties (such as soil moisture, oxygen concentration and soil nitrogen content) to affect soil nitrogen turnover, so as to promote the change of isotopic value [30]. On the whole, these studies regarding the pattern of δ^15^N values have greatly increased our understanding of ecosystem N-cycling.

Nonetheless, we still know little about how co-varying climatic and edaphic variables independently affect soil δ^15^N values along environmental gradients due to the following two reasons. First, because nitrogen cycle and the factors affecting soil δ^15^N values are very complex, the climate and soil variables determining the patterns of soil δ^15^N probably involve direct and indirect influence paths on a regional scale. For instance, low δ^15^N values usually occur in ecosystems with high precipitation and high carbon contents, but with low temperature and low N concentrations [15,25]. In other words, all the influences among the climate and soil factors will form a network, and a path analysis can disclose how these factors interact to affect soil δ^15^N values. However, most of the existing studies focused on the overall impact of single factor on soil nitrogen isotope without distinguishing the direct and indirect effects or failed to estimate the causal relationships among the variables. Second, most of the existing studies primarily used bivariate linear regression or multiple regressions to determine how climatic and edaphic variables influence soil δ^15^N values at different spatial scales. Such methods cannot evaluate the interplay of environmental factors on soil δ^15^N, and also fail to determine the extent to which variables explain the δ^15^N values owing to the covariation of environmental factors [21,31]. This may also be one of the reasons for the relatively low reliability of explaining ecosystem nitrogen cycle through δ^15^N signal in the existing studies.

Temperate grasslands in northern China account for approximately 12% of global grassland area and provide crucial ecosystem services. In particular, grassland soils have great potential to regulate biogeochemical cycle under the background of global climate change [32]. Therefore, it is vital to investigate the impact mechanisms of environmental factors on soil δ^15^N along temperate grassland for elucidating soil N-cycling on regional scale [20,33]. In this study, we use a path analysis based on partial least squares regression (PLS-PATH) to assess the direct and indirect impact of environment factors on soil δ^15^N values in the temperate grasslands in northern China and causal relationships among the variables. The research strives to answer the following three questions. First, what are the direct and latent indirect influences of the climate and soil variables on variance of soil δ^15^N value? Second, how do the causal path relationships between driving factors? Third, what is the order of all variables’ influences on soil δ^15^N with respect to their influential intensity?

## Materials and methods

### Study transect and field sampling

The study was conducted along a 1200 km transect across the temperate grasslands of northern China. The east-west transect covered longitudes from 112°53′ to 121°98′E and latitudes from 42°12′ to 43°98′N, with elevations between 158 and 1406m. The transect was characterized predominantly by a temperate continental monsoon climate, with a MAT gradient from 1.71 to 7.10°CC and a MAP gradient from 154 to 446 mm, respectively.

Along the transect from east to west, the main vegetation types are temperate meadow grassland, typical temperate grassland and temperate desert [34]. The soils related to these three grassland types were chernozem, chestnut soil and aeolian sand soil, respectively, which were the same substrate age [35]. The soil pH values of the 0~20cm layer varied between 6.2 and 8.5 [26]. With such climatic and edaphic gradients, this transect provides an ideal place for examining the direct and indirect effects of environmental factors on soil δ^15^N and the interrelationships between driving factors.

Our studies were approved by the Forestry and Grassland Bureau of Inner Mongolia Autonomous Region and by the Institute of Grassland Research of CAAS. During the period from July to August in 2017, a total of 40 undisturbed sites were chosen to collect soil samples along this transect. At every site, five 1 m **×**1 m plots within an area of 10 m **×** 10 m were established for soil sampling. Within each plot, five soil cores with a diameter of 2.5 cm were randomly taken at 0 to 20cm depth. A total of 25 soil cores were obtained from each site and fully mixed as a composite sample. After removing fine roots and other coarse materials in soils, each composite sample was sieved by a 2.0 mm screen and then divided into two parts: one was stored in an incubator (at 4°C) for determining the physicochemical properties of the soil (e.g., soil C and N contents, soil clay and pH), and the other was air-dried and ground to uniformity with a ball mill (NM200, Retsch, Hann, Germany) for measurement of soil δ^15^N value. Additionally, the altitude, longitude and latitude of sampling sites were determined by Global Positioning System. The MAT and MAP of each sampling site were obtained from Data Center for Resource and Environment Science in China (https://www.resdc.cn/Default.aspx), China Meteorological Data Service Centre (http://data.cma.cn), and from local weather stations. Further detailed information about the sampling sites was summarized in Table 1.

**Table 1:** The general information of the sampled sites in the study area. MAT and MAP in Table 1 are the abbreviations of mean annual temperature and mean annual precipitation, respectively (the same below). These climate data were the averages of observation data collected during a 30 year period (1987–2017). TMS, TTS and TD represent temperate meadow steppe, typical temperate steppe and temperate desert, respectively. The dominant soil types were from the “1:1,000,000 Soil Map of China in 2007”.

Sample sites	Longitude(°E)	Latitude(°N)	Altitude(m)	MAT (°C)	MAP (mm)	Soil type	Vegetation type
Tongliao1	121.96	43.63	202	6.81	387	Chernozem	TMS
Tongliao2	121.97	43.61	207	5.92	446	Chernozem	TMS
Tongliao	121.98	43.60	205	5.90	442	Chernozem	TMS
Kailu1	121.95	43.73	158	6.62	375	Chernozem	TMS
Kailu2	121.89	43.57	184	7.10	396	Chernozem	TMS
Kailu3	121.58	43.74	164	6.76	408	Chernozem	TMS
Arhorqinqi1	120.56	43.74	342	6.06	359	Chestnut soil	TTS
Arhorqinqi2	120.75	43.66	281	6.49	358	Chestnut soil	TTS
Arhorqinqi3	120.64	43.63	285	6.53	358	Chestnut soil	TTS
Bairinzuoqi1	119.36	43.88	486	5.43	339	Chestnut soil	TTS
Bairinzuoqi2	119.40	43.89	472	5.26	353	Chestnut soil	TTS
Bairinzuoqi3	119.23	43.58	477	5.31	384	Chestnut soil	TTS
Bairinyouqi1	119.99	43.40	698	4.14	362	Chestnut soil	TTS
Bairinyouqi2	119.62	43.45	520	4.98	334	Chestnut soil	TTS
Bairinyouqi3	119.71	43.44	453	5.34	329	Chestnut soil	TTS
Linxi1	118.19	43.61	758	4.19	341	Chestnut soil	TTS
Linxi2	118.54	43.53	648	4.86	339	Chestnut soil	TTS
Linxi3	118.71	43.58	664	4.68	347	Chestnut soil	TTS
Kesketonqi1	117.71	43.47	1346	2.62	365	Chestnut soil	TTS
Kesketonqi2	117.84	43.39	1159	2.65	351	Chestnut soil	TTS
Kesketonqi3	117.86	43.53	1050	3.42	347	Chestnut soil	TTS
Xilin east1	117.09	43.31	1267	1.75	398	Chestnut soil	TTS
Xilin east2	117.32	43.30	1269	1.71	408	Chestnut soil	TTS
Xi Ujimqin1	117.82	43.87	1168	2.23	230	Aeolian sand soil	TD
Xi Ujimqin2	117.09	43.85	1047	2.87	219	Aeolian sand soil	TD
Xi Ujimqin3	117.64	43.95	1076	2.48	244	Aeolian sand soil	TD
Xilinhot1	116.40	43.77	1099	2.28	324	Chestnut soil	TTS
Xilinhot2	116.54	43.71	1200	1.74	350	Chestnut soil	TTS
Xilinhot3	116.60	43.76	1150	1.97	342	Chestnut soil	TTS
Abagqi1	115.01	43.98	1120	2.13	265	Chestnut soil	TTS
Abagqi2	115.65	43.93	1133	2.02	295	Chestnut soil	TTS
Abagqi3	115.28	43.93	1164	1.91	286	Chestnut soil	TTS
Baiqi1	115.13	42.24	1406	2.06	363	Chestnut soil	TTS
Baiqi2	115.12	42.12	1405	2.03	364	Chestnut soil	TTS
Sonidzuoqi1	114.09	43.85	1047	2.37	219	Aeolian sand soil	TD
Sonidzuoqi2	114.47	43.92	1022	2.57	228	Aeolian sand soil	TD
Sonidzuoqi3	114.82	43.98	1131	2.09	259	Aeolian sand soil	TD
Erlanhot1	112.53	43.67	998	3.64	160	Aeolian sand soil	TD
Erlanhot2	112.73	43.70	987	3.63	154	Aeolian sand soil	TD
Erlanhot3	112.80	43.73	960	3.74	160	Aeolian sand soil	TD

### Measurements of soil δ^15^N and soil properties

The δ^15^N value of soil sample was measured by a Finnigan Delta^Plus^XP (Thermo scientific, Waltham, Massachusetts, USA) coupled with an automatic elemental analyzer (Flash EA1112, Thermo Finnigan). The isotope result of soil N was calculated with the following equation: δ^15^N (‰) = (*R*_sample_/*R*_standard_ -1)×1000, where *R*_sample_ and *R*_standard_ represent the ratios of ^15^N/^14^N in the sample standard. The standard of δ^15^N is atmospheric N_2_. The standard deviation of above isotopic repeated measurements was ±0.15‰.

Soil pH value was determined using a pH meter (HI-9125, Hanna Instruments Inc, Woonsocket, RI) with a dry soil-water ratio of 1:2.5. Total organic carbon (TOC) and nitrogen (TN) concentrations of soil samples were measured by a TOC/TN analyzer (Multi N/C 3100CLD, Jena, Germany). Soil C/N ratio was expressed by the quotient of TOC and TN content. Another subsample was separated into clay (<2μm) by ultrasonic energy method. The result of particle size analysis was expressed as a percentage of the weight of oven-dried soil. The values of all the above observed variables, which includes the MAP and MAT of sampling locations, soil N, soil pH, soil C: N, soil clay and soil δ^15^N, were listed in S1 Appendix.

### PLS-PATH analyses

Before PLS-PATH analyses, all data were tested for normality and standardized to eliminate the influence caused by different variable units. The potential relationship between various variables (MAP, MAT, soil N, soil C/N, pH and soil clay) and soil **δ**^15^N was analyzed by bivariate correlations and linear regressions. The normality test results of the variables showed that the absolute values of critical ratios were greater than 0.05, indicating that the kurtosis (or skewness) coefficients of all variables were significant (Table 2). This also meant that it was suitable for using path analysis in our study. Moreover, the correlation analysis results showed that there was obvious collinearity among some independent variables (Table 3), which means that the indirect impact is also the driver of the soil **δ**^15^N in ecosystem. To eliminate collinearity issues among the variables and address how climatic and edaphic variables interactively affect soil **δ**^15^N, a PLS-PATH analysis was used to explore the direct and indirect influence of each environmental variable on soil **δ**^15^N values and prospective causal relationships among variables at the regional scale. PLS-PATH analysis is considered as a special form of PLS-SEM [35]. Because of the lax requirements of data distribution and small sample size, it has become one of the most popular approaches for estimating complex path relationships. In the PLS-PATH analyses, the direct influence of variables on soil **δ**^15^N as well as indirect and total impacts of variables on soil **δ**^15^N can be expressed by direct, indirect and total path coefficients, respectively. At the same time, the causal relationship between variables can be shown by vectors in the path diagram. On the whole, PLS-PATH analysis is more comprehensive and accurate compared with simple regression analysis. The basic idea of PLS-PATH analysis is as follows: first of all, assume that the explained variable *y* has several explanatory variables *x*_i_ (*i* = 1, 2, 3,…, n), and the relationship between each explanatory variable *x*_i_ and *y* is linear. Second, PLS regression is used to establish the optimal regression equation between explanatory variables and explained variable. Thereupon, the path coefficient $P_{x_{i},y}$ of the explanatory variable ***x***_***i***_ to the explained variable **y** in the PLS regression equation can be decomposed into direct path coefficient (denoted as ${DP}_{x_{i},y}$) and indirect path coefficient (namely, the action coefficient of explanatory variable ***x***_***i***_ to explained variable **y** through explanatory variable ***x***_***j***_, denoted as ${IP}_{x_{i},x_{j},y}$). The calculation formulas of direct action coefficient, indirect action coefficient and total action coefficient of independent variable on dependent variable are as follows:

$$P_{x_{i},y}=r_{x_{i},y}\times (S_{x_{i}}/S_{y})$$ (1)

$${IP}_{x_{i},x_{j},y}=r_{x_{i},x_{j}}\times r_{x_{i},y}\times (S_{x_{i}}/S_{y})$$ (2)

$$P_{x_{i},y}={DP}_{x_{i},y}+\sum_{j\neq i}^{n-1}{IP}_{x_{i},x_{j},y}$$ (3)

**Table 2:** Statistical description and normality test of all the variables in the study transect. The critical ratio is the skewness (or kurtosis) of each variable divided by its standard error.

Variables	Min	Max	Mean	Standard error	Skewness	Critical ratio	Kurtosis	Critical ratio
Soil δ^15^N	2.54	7.49	5.13	1.14	-0.13	-0.11	-0.35	-0.31
MAP	153.5	446.0	324.5	32.9	1.83	0.06	-1.89	-0.06
MAT	1.71	7.10	3.91	1.78	0.35	0.20	-1.40	-0.62
pH	6.70	8.65	7.73	0.46	-0.27	-0.59	-0.44	-0.96
Soil N	0.07	1.76	0.93	0.44	0.29	0.66	-0.93	-2.11
Soil clay	16.9	28.00	21.19	3.77	0.54	0.14	-1.14	-0.30
Soil C:N	10.66	20.90	16.89	2.44	-0.17	-0.07	-0.16	0.07

**Table 3:** Pearson’s correlation coefficients for climatic and edaphic variables in explaining soil δ^15^N.

Variables	MAP^a^	MAT^b^	Soil pH	Soil N	Soil clay	soil C:N	Soil δ^15^N
MAP	1.000						
MAT	0.417**	1.000					
Soil pH	-0.256	0.453**	1.000				
Soil N	-0.598**	-0.533**	-0.234	1.000			
Soil clay	-0.540**	-0.116	0.007	0.293	1.000		
Soil C:N	0.153	0.262	0.293	-0.167	-0.277	1.000	
Soil δ^15^N	-0.662**	-0.639**	-0.100	0.547**	0.402*	-0.385*	1.000

* and ** indicate that the correlation is significant at the levels of 0.05 and 0.01 (two-tailed), respectively.

Where $r_{x_{i},y}$ represent the partial correlation coefficient of *x*_*i*_ and y; $r_{x_{i},x_{j}}$ denotes the simple correlation coefficient of *x*_*i*_ and *x*_*j*_; ${S_{x}}_{i}$ and *S*_*y*_ are the standard deviations of the explanatory variable *x*_*i*_ and the explained variable *y*, respectively. Basic statistics of the data was conducted with SPSS 18.0, and path coefficients were estimated via the maximum likelihood method using SmartPLS V3.2.8 software [36].

## Results

### Path coefficients and decomposition results of climatic and edaphic variables on soil δ^15^N values

**Fig 1:**
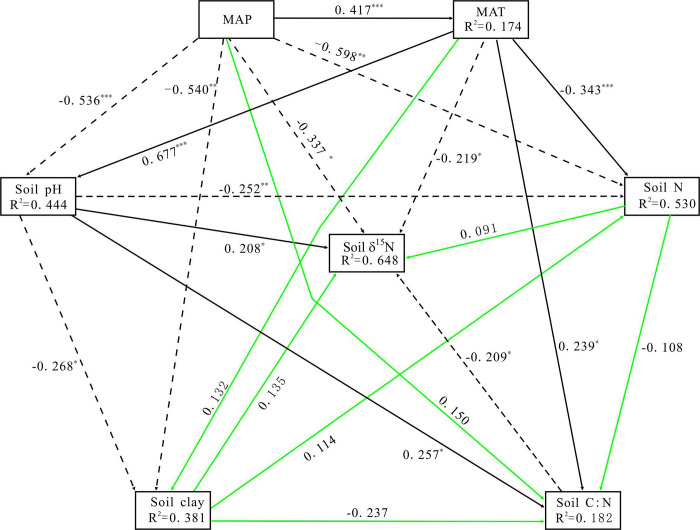
Diagram of path analysis. The PLS-PATH analysis was used to explore the direct and indirect effects of each variable on soil δ^15^N value, as well as the causal relationship between variables. The number adjacent to arrow is standardized path coefficients, showing the influence size of the relationship; Continuous and dotted black arrows denote positive and negative relationship, and green lines indicate no significant relationship. *, ** and *** represent significant differences at the levels of 0.05, 0.01 and 0.001, respectively.

A path diagram that displays the variable relationships was established based on the PLS-PATH analysis. Fig 1 showed that, along the transect in temperate grassland of northern China, climatic and edaphic variables explained 64.8% of the total variance in soil δ^15^N values. MAP and MAT together with soil C/N directly determined soil δ^15^N values, and the three had a significant negative effect on soil δ^15^N values. On the contrary, soil clay, soil pH and soil N content had a positive effect. PLS-PATH analyses further indicated that among all independent variables, MAP, MAT and soil pH could significantly and directly alter other climate and/ or soil variables (soil N, soil clay and soil C/N) (Fig 1). As can be seen from the decomposition results of the total action coefficient of each variable (Table 4), MAP, MAT and soil C/N ratio had a total negative effect (direct and indirect) on soil δ^15^N values, and the standardized total effect of these variables on soil δ^15^N values was -0.677, -0.229 and -0.209 correspondingly; while soil N, soil clay and soil pH had positive total effects on soil δ^15^N values, and the standardized total effect of them was 0.114, 0.198 and 0.072, respectively. This showed that soil δ^15^N displayed a large increase with soil N, soil clay and pH across temperate grassland in northern China. Additionally, their total effects (expressed in absolute value, the same below) were, in proper order, MAP > MAT > soil C/N > soil clay > soil N > soil pH. Meanwhile, the MAP, MAT and soil C/N had a direct negative influence on soil δ^15^N, and their standardized direct effects were -0.337, -0.219 and -0.209, respectively. The sequence of direct influence of all factors on soil δ^15^N values was MAP > MAT > soil C/N > soil pH >soil clay > soil N, and the indirect influence of all factors on soil δ^15^N values was, in order, MAP >soil pH > soil clay > soil N > MAT> soil C/N.

**Table 4:** The decomposition results of the path analysis. Through PLS-PATH analysis, the total impact of each variable on soil δ^15^N is divided into direct and indirect effects. The negative sign indicates that the variable makes the soil δ^15^N decrease.

Causal variables	Result variables	Total effects	Direct effects	Indirect effects
MAP	Soil δ^15^N	-0.677	-0.337	-0.340
MAT	Soil δ^15^N	-0.229	-0.219	-0.010
Soil N	Soil δ^15^N	0.114	0.091	0.023
Soil pH	Soil δ^15^N	0.072	0.208	-0.136
Soil C: N	Soil δ^15^N	-0.209	-0.209	0.000
Soil clay	Soil δ^15^N	0.198	0.135	0.063

### Influence mechanism of climatic and edaphic variables on soil δ^15^N values

**Fig 2:**
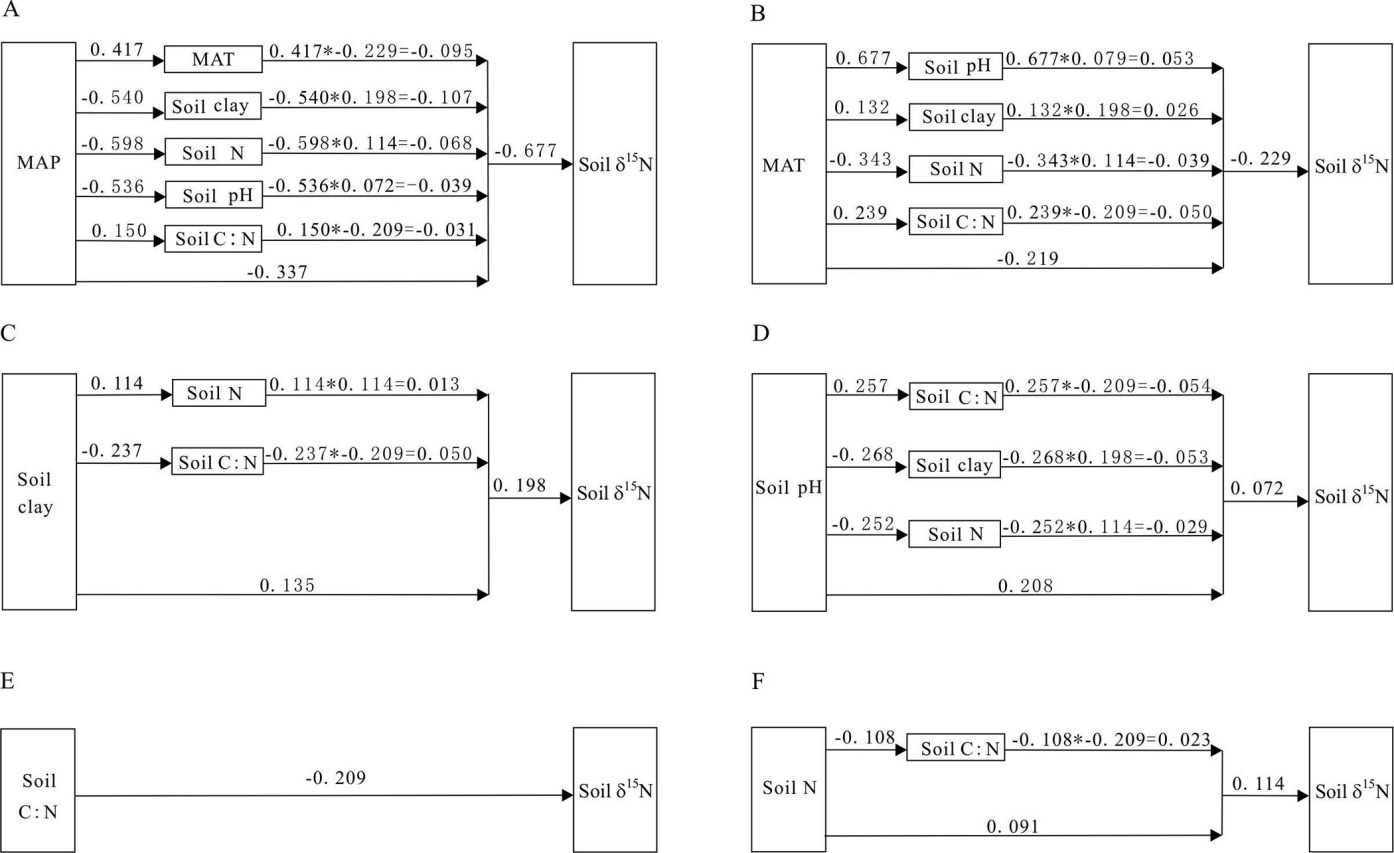
Influence path of climate and soil factors on soil δ^15^N. (A) The paths of MAP on soil δ^15^N. (B) The paths of MAT on soil δ^15^N. (C) The paths of soil clay on soil δ^15^N. (D) The paths of soil pH on soil δ^15^N. (E) The path of soil C: N on soil δ^15^N, yet no indirect effect of soil C: N ratio on soil δ^15^N was found. (F) The paths of soil N on soil δ^15^N.

Fig 2 described the direct and indirect effects of various factors on soil δ^15^N. As could be seen from Fig 2A, soil δ^15^N was very sensitive to both direct and indirect impacts of MAP. When MAP increased by 1 standard deviation, the soil δ^15^N directly decreased by 0.337 standard deviation. Meanwhile, the indirect effect of MAP on soil δ^15^N through MAT, soil clay, soil N, soil pH and soil C: N was -0.340 (Table 4). As shown Fig 2B, the contribution of MAT to soil δ^15^N mainly came from its direct impact, and the direct impact of MAT on soil δ^15^N was -0.219. In addition, MAT had a negative indirect effect on soil δ^15^N through soil N and C:/N ratio; whereas exhibited a positive indirect effect on soil δ^15^N through soil pH and soil clay. Similarly, following Fig 2C, the contribution of soil clay to soil δ^15^N mainly was from its direct effect. The total indirect impact of clay content on soil δ^15^N was fairly small (0.063). Among them, the indirect influence of soil clay on soil δ^15^N through soil N was 0.013, while the indirect influence through soil C/N ratio was 0.050. The direct contribution of soil pH increasing soil δ^15^N was 0.208, and the indirect impacts of soil pH through soil C/N, clay content and soil N were -0.054, -0.053 and -0.029 respectively (Fig 2D), amounting to -0.136 (Table 4).

δ

The soil C/N ratio had no indirect effect on soil δ^15^N (Table 4). The increase of soil C/N directly leaded to significant decrease of soil δ^15^N. When the soil C/N ratio increased by 1 standard deviation, the soil δ^15^N directly decreased by 0.209 (Fig 2E). As shown in Fig 2F, soil N had a positive direct impact on soil δ^15^N with influence strength of 0.091. However, the indirect impact of soil N on soil δ^15^N through soil C/N was quit small (0.023).

## Discussions

Many scholars have studied how climate and soil factors influence on soil δ^15^N. The study used the PLS-PATH method to estimate the direct and indirect impact of various factors on soil δ^15^N and their causal relationships. Some of our results confirmed previous conclusions. On the whole, climatic factors had stronger effects than those of edaphic factors on soil δ^15^N values (Table 4). The total impact strength of climate factors on soil δ^15^N was 0.90 (in absolute value); whereas the total impact intensity of edaphic factors on soil δ^15^N was 0.574 (in absolute value). This indicated that the soil δ^15^N value was more susceptible to changes of climatic factors than to those of edaphic factors. The following was an analysis of how various climatic and edaphic factors control soil δ^15^N.

### Climatic controls on soil δ^15^N

**Fig 3:**
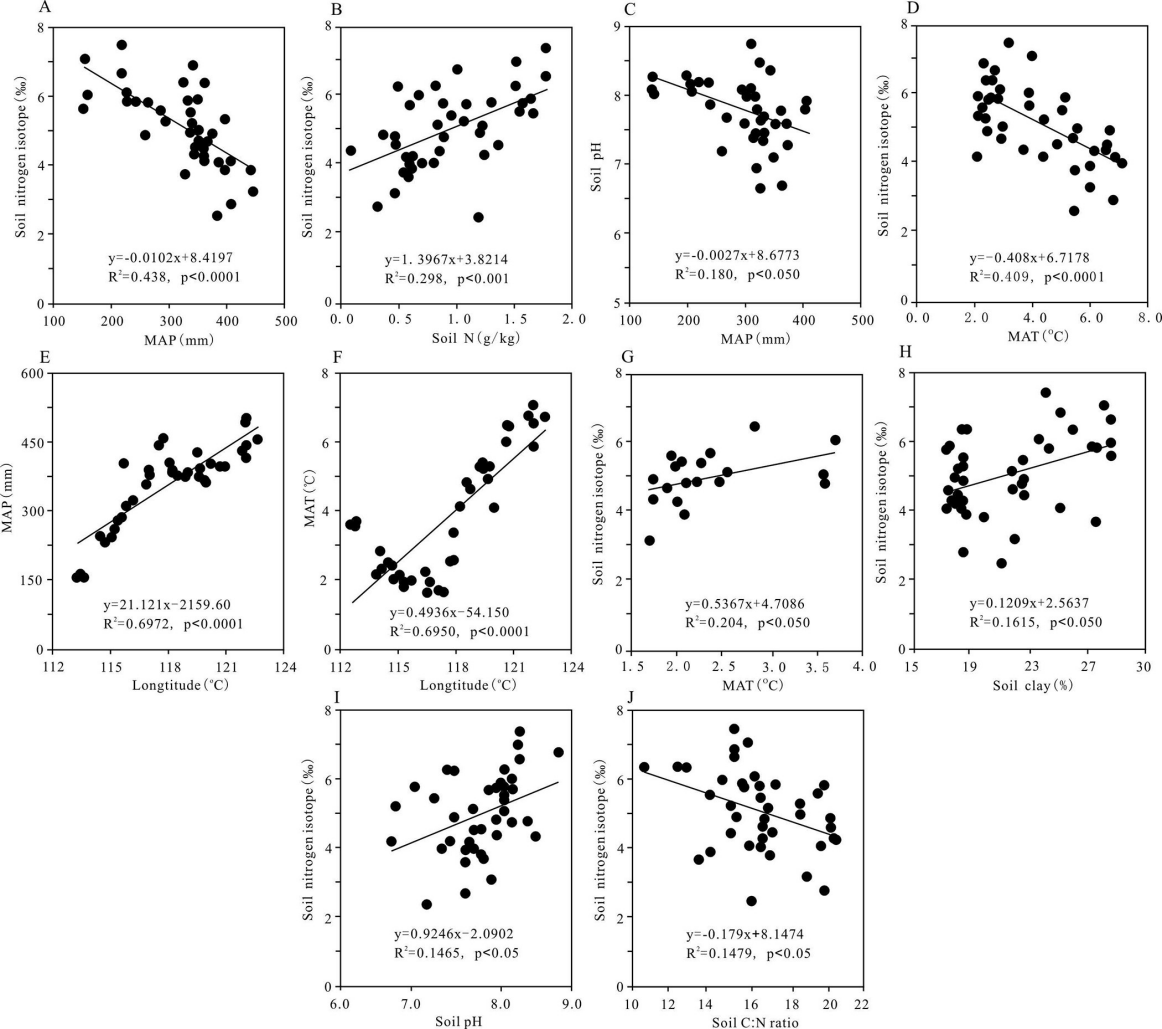
Changes of soil δ^15^N with various factors and the relationships between some factors. The linear regression method was used to analyze how the soil δ^15^N changes with various environmental factors (MAP, MAT, soil N, soil C/N, pH, soil clay and longitude) and the relationships between some factors. (A) Change of soil δ^15^N with the MAP. (B) Change of soil δ^15^N with the soil N. (C) Change of soil pH with the MAP. (D) Change of soil δ^15^N with the MAT. (E) Change of the MAP with the longitude. (F) Change of the MAT with the longitude. (G) Change of soil δ^15^N with the MAT at the western part of the transect. (H) Change of soil δ^15^N with the soil clay. (I) Change of soil δ^15^N with the soil pH. (J) Change of soil δ^15^N with the soil C:N ratio.

Many scholars generally think that the change of soil δ^15^N is mainly determined by the isotopic values of input N and output N. Since the δ^15^N of input N is close to zero and usually lower than that of soil, the N output process may leave more ^15^N in soil compared with the input [8,11]. In our study, soil δ^15^N decreased significantly with increasing MAP along the transect (Fig 3A), which is in accordance with the results obtained by other scholars [13,15,37,38]. The strong negative effect of MAP on soil δ^15^N in temperate grassland of northern China may be caused by the following two reasons. First, the openness of nitrogen cycle is determined by precipitation and decreases with precipitation. Thus, the more open N-cycle in arid environment compared to that in wet environment would likely lead to disproportionate loss of inorganic N through NH_3_ volatilization (the N loss caused by leaching and denitrification is generally very small due to the low soil moisture content in our study area) during nitrification process, which causes a greater ^15^N-enrichment in soil organic N and an increase of soil δ^15^N [39,40]. Second, precipitation is one of the most critical determinants of N cycle in arid and semi-arid areas. The N utilizing efficiency by plants may increase with MAP increasing when soil water content is unsaturated and remains aerobic, which results in more ^15^N-depleted in the soil pool. Meanwhile, as precipitation increase, the incomplete decomposition of plant residue may bring more enriched ^14^N organic matter to soil N pool, thus causing soil δ^15^N values to be lower [18]. Besides that, MAP can indirectly affect soil δ^15^N through its effects on other factors (Fig 2A). For example, when the MAP increases by 1 standard deviation, the soil N will decrease by 0.598 standard deviation, which leads to a reduction of soil δ^15^N owing to the positive correlation between soil δ^15^N and soil N (Fig 3B). For another instance, precipitation usually affects the leaching of alkaline cations in soil, so as to affect the soil pH value. Since soil pH value is negatively correlated with MAP across the transect (Fig 3C), the relatively high soil pH in the dry sites is easy to accelerate the volatilization of NH_3_, resulting in positive soil δ^15^N values [15,41]. But, remarkably, the indirect effect strength of MAP on soil δ^15^N values is different because of different relative importance of driving factors.

Most studies show that soil δ^15^N values usually increase with MAT [15,26,27,37]. However, the relation between soil δ^15^N and MAT in temperate grassland of northern China was clearly inconsistent with those obtained from other regions and globally. We argued that the negative soil δ^15^N-MAT relationship (Fig 3D) may be owing to these two reasons. First, such a pattern was possibly related to the narrow temperature range (MAT: 1.71 to 7.10°CC) along the study transect. As is known to all, the shift in N cycling from organic or ammonium-dominated status to nitrate-dominated status is usually considered as the potential control of soil δ^15^N [42]. Accordingly, compared to the wide temperature range obtained from global and other regional scales, the narrow range of MAT across our study area was insufficient to cause a large impact on the N-cycling process and, thus, on affecting soil δ^15^N values [18]. Second, the negative correlation of soil δ^15^N with temperature may also be due to simultaneous effect of rain and heat. In our study area, approximately 60% to 80% of annual precipitation occurs in summer season [34]. Although increasing temperature can accelerate the rates of soil organic N mineralization and NH_3_ volatilization, resulting in accelerated ^14^N loss and positive soil δ^15^N values, due to the synchronization of rain and heat at the eastern part of the transect (Fig 3E and 3F), increasing precipitation can contribute more negative soil δ^15^N values in this region, thus masking the positive correlation between soil δ^15^N and MAT to a certain extent [18]. This result was supported by the indirect effect of MAP on soil δ^15^N through MAT (Fig 2A). To examine whether there is a positive relation between soil δ^15^N and MAT, we extracted 19 sites data at the western part of the transect, and found that soil δ^15^N of these sites increased significantly with the increase of MAT (Fig 3G). Furthermore, PATH analyses showed that MAT indirectly influenced soil δ^15^N through edaphic factors (Fig 2B). For instance, the standardized indirect influence of MAT on soil δ^15^N through soil N is -0.039, demonstrating that temperature also has an indirect negative effect on soil δ^15^N. This result confirms the view that the soil N retention rate is lower in high-temperature environments than in low-temperature regions [24], and also reflects the apparent temperature-dependence of soil N cycle processes [4].

### Edaphic controls on soil δ^15^N

Besides climate, our PATH analyses also showed that edaphic factors play important roles in controlling soil δ^15^N values. In the current study, soil δ^15^N values exhibited a significant increase with soil clay across temperate grasslands in northern China (Fig 3H). The positive correlation between soil δ^15^N and clay may be directly driven by a larger proportion of discriminating gaseous N (N_2_O and NO) losses from soil with high clay concentrations. As a result, the large amount of gaseous N loss may bring about higher δ^15^N values in fine-textured soils because they have a great potential impact on ^15^N/^14^N fractionation [17,30]. Despite all this, the increase of soil δ^15^N with clay content is also inseparable from the indirect effect of soil clay (Fig 2C). That is because soil clay can influence soil N concentration and soil C: N ratio through its fixing more ^15^N-enriched soil organic matter [43]. Due to the greater decomposition of organic matter in soil with high clay concentration, the soils in warm and /or dry environments may contain soil organic matter with high δ^15^N because mineral-associated organic matter contains a greater proportion of N.

In our study area, soil δ^15^N exhibits a significant upward trend with soil pH values along the transect (Fig 3I), suggesting that soil pH has an important impact on ecosystem N cycle. The increase of soil δ^15^N with soil pH value might be caused by the increase of NH_3_ volatilization under alkaline conditions [26]. Some experimental evidences show that soil pH can influence internal N cycle by affecting microbial nitrification and denitrification, and also control gaseous N loss via NH_3_ volatilization because at high pH, the ammonium-ammonia equilibrium is inclined to the gaseous form, which enriches soil N with ^15^N [28,30]. Moreover, soil pH indirectly influences soil δ^15^N through influencing soil C: N ratio, soil clay, and soil N (Fig 2D). The total indirect effect strength of soil pH on soil δ^15^N is -0.136, indicating that soil pH is also a main indirect driver of nitrogen isotope fractionation.

In our regional scale, soil δ^15^N decreased significantly with the increase of soil C/N ratio along the transect (Fig 3J), and this total effect of soil C/N was all from its direct effect (Fig 2E). There are reports that soil C: N ratio can greatly influence the operation of N cycle process at regional scale [27,44]. Therefore, the reduction of soil δ^15^N with soil C: N ratio may be due to its impacts on soil gaseous N losses. It was reported that soil N_2_O emissions exhibited an exponential decline as soil C/N ratio increased [45]. The significant negative correlation of soil δ^15^N and C: N ratio indicates that a lower C: N ratio will enhance soil gaseous losses in the process of soil mineralization, nitrification and denitrification, and thus cause larger soil ^15^N enrichment. And it also means that the loss of gaseous N can regulate soil δ^15^N in temperate grassland of northern China.

Besides, some scholars found that soil δ^15^N increased or decreased as soil N contents increased [26,27], but we observed a significant positive correlation between soil δ^15^N and soil N across the transect (Fig 3B), which was consistent with the results previously reported by Shan et al. [44] on the Chinese Loess Plateau. The positive relationship between soil δ^15^N and soil N might result from the increase in the fractionation of ^15^N with the increase of soil N reserves. Normally, the ^15^N abundance of N input is close to zero and lowers than soil ^15^N. Therefore, N outputs and the fractionation of microbial activities in soils may have great influence on soil δ^15^N [20,46,47]. In this study, with the reduction of MAP and MAT from east to west along the transect (Fig 3E and 3F), warm and dry sites may have led to increase in microbial activity relative to cold and wet sites [35]. In the case of high soil N content, isotope depleted ^14^N is preferentially lost from the soil through NH_3_ volatilization and microbial denitrification, resulting in the enrichment of ^15^N in soil pool and subsequent increases in soil δ^15^N. Furthermore, soil N indirectly affected soil δ^15^N by affecting soil C/N, but the influence intensity is small (Fig 2F).

## Conclusions

In short, a regional-scale soil samples were collected along an environmental gradient in temperate grassland of northern China. Four conclusions emanate from PLS-PATH analysis conducted herein, which will provide valuable information for the response of ecosystem N cycle to climatic and edaphic changes.

(1) Soil N, soil pH, and soil clay have positive total effect on soil δ^15^N, whereas MAP, MAT, and soil C/N ratio have negative total effect on soil δ^15^N. The total influence is, in order, MAP > MAT > soil C: N ratio > soil clay > soil N > soil pH (in absolute value). Among them, soil pH, soil clay, and soil N have a positive direct effect on soil δ^15^N. In addition, the indirect impact of all factors on soil δ^15^N is, in order, MAP > soil pH > soil clay > soil N > MAT > soil C/N. (2) Across the transect, MAP exerted the first-order controls on soil δ^15^N with negative correlation, and directly or indirectly influenced soil δ^15^N value through affecting MAT, soil clay, soil pH, soil N and C/N ratio. Soil δ^15^N values significantly decreased as MAT increased. The negative soil δ^15^N-MAT relationship, which is in contrast with previous research, may be due to the narrow temperature range and simultaneous effect of rain and heat in the study area. (3) Soil C: N ratio imposed a significant negative direct effect on soil δ^15^N. This result indicates that in temperate grassland, soil C/N ratio can regulate soil δ^15^N values by gaseous N loss processes. Soil clay, soil N content, and soil pH have a total positive effect on soil δ^15^N. However, soil pH has a significant negative indirect influence on soil δ^15^N through its effects on soil clay, soil N and C: N ratio. Consequently, the total positive impact of soil pH on soil δ^15^N is weak. (4) These results suggest that climatic and edaphic factors are to some extent coupled in controlling soil ^15^N abundance. It should be noted that the current research did not consider plant species and microbial activities as the factors affecting soil δ^15^N.

## Supporting information

S1 Appendix Geospatial, soil and climatic data. All observed data are available, which includes the longitude and latitude of sampling locations, mean annual precipitation (MAP), mean annual temperature (MAT), altitude, soil N, soil pH, soil C:N, soil clay and soil δ^15^N.
(PDF)
