# Supplementary material for: Climatic and edaphic controls over soil δ15N in temperate grassland of northern China: A PLS-PATH analysis
Source: PLoS One. 2022 Oct 31;17(10):e0265795. doi: 10.1371/journal.pone.0265795 (PMC9621419; doi:10.1371/journal.pone.0265795)
Supplement: S1 Appendix — All observed data are available, which includes the longitude and latitude of sampling locations, mean annual precipitation (MAP), mean annual temperature (MAT), altitude, soil N, soil pH, soil C:N, soil clay and soil δ15N. (PDF) [file pone.0265795.s001.pdf]

Data for: Climatic and edaphic controls over soil $\delta^{15}N$ in temperate grassland of northern China: A PLS-PATH analysis

S1 Appendix. Geospatial, soil and climatic data. All observed data are available, which includes the longitude and latitude of sampling locations, mean annual precipitation (MAP), mean annual temperature (MAT), altitude, soil N, soil pH, soil C:N, soil clay and soil $\delta^{15}N$.

location	latitude(^{O}N)	Longitude(^{O}E)	Altitude(m)	MAP(mm)	MAT (^{O}C)	pH	Soil N(g/ kg)	Soil C:N	Soil clay (%)	Soil δ^{15}N (‰)
sonidzuoqi1*	43.85	114.09	1047	219.0	2.37	8.30	1.76	15.5	28.0	6.68
sonidzuoqi2*	43.92	114.47	1022	228.0	2.57	8.20	0.66	16.6	23.1	6.12
sonidzuoqi3	43.98	114.82	1131	259.0	2.09	8.20	0.88	17.1	22.0	4.89
Abagqi1*	43.98	115.01	1120	265.0	2.13	7.90	0.58	16.0	16.9	5.83
Abagqi2*	43.93	115.65	1133	295.0	2.02	7.70	0.82	15.3	17.7	5.27
Abagqi3	43.93	115.28	1164	286.0	1.91	7.20	1.65	14.2	18.0	5.58
Kesketonqi1*	43.47	117.71	1346	365.0	2.6	7.70	1.35	20.7	17.0	4.66
Kesketonqi2*	43.39	117.84	1159	351.0	2.65	7.45	1.19	19.0	17.5	5.02
Kesketonqi3	43.53	117.86	1050	347.0	3.42	7.40	1.23	17.0	17.3	4.34
Erlanhot1*	43.67	112.53	998	153.5	3.64	8.10	1.53	20.0	28.0	5.64
Erlanhot2*	43.70	112.73	987	159.5	3.63	8.05	1.63	14.9	28.0	6.02
Erlanhot3	43.73	112.80	960	155.8	3.74	8.28	1.51	16.2	27.5	7.09
Xi Ujimqin banner*	43.87	117.82	1168	230.0	2.23	8.10	1.56	20.3	17.0	5.87
Xi Ujimqin banner*	43.85	117.09	1047	219.0	2.87	8.30	1.76	15.5	23.6	7.49
Xi Ujimqin banner	43.95	117.64	1077	244.0	2.48	8.20	1.07	16.9	23.8	5.85
xilinhot1*	43.77	116.40	1099	324.0	2.28	8.10	0.81	12.5	25.4	6.39
xilinhot2*	43.71	116.54	1200	350.0	1.74	6.95	1.29	15.9	26.7	5.91
xilinhot3	43.76	116.60	1150	342.0	1.97	8.80	0.99	15.5	24.6	6.89
Linxi1*	43.61	118.19	758	341.0	4.19	8.10	1.21	17.3	21.3	5.22

Linxi2*	43.53	118.54	648	339.0	4.86	8.10	0.94	16.9	22.0	5.52
Linxi3	43.58	118.71	664	347.0	4.68	8.00	0.07	17.5	22.1	4.51
xilin east1*	43.31	117.09	1267	398.0	1.75	6.70	1.05	19.0	18.0	5.34
xilin east2	43.30	117.32	1269	408.0	1.71	7.30	0.69	20.1	16.9	4.13
Bairinyouqi*	43.40	119.99	698	362.0	4.14	7.70	0.79	16.3	24.6	4.13
Bairinyouqi*	43.45	119.62	520	334.0	4.98	8.00	0.87	17.7	27.0	5.88
Bairinyouqi	43.94	119.71	453	329.0	5.34	7.60	0.57	13.6	27.0	3.74
ArHorqinqi1*	43.74	120.56	342	359.0	6.06	7.65	0.55	20.9	17.5	4.31
ArHorqinqi2*	43.66	120.75	281	358.0	6.49	6.65	0.61	20.8	18.0	4.34
ArHorqinqi3	43.63	120.64	285	358.0	6.53	8.50	0.84	15.3	17.6	4.49
kailu1*	43.73	121.95	158	375.0	6.62	8.40	0.45	20.6	18.0	4.92
kailu2*	43.57	121.89	184	396.0	7.10	7.80	0.60	14.2	18.2	3.96
kailu3	43.74	122.58	164	408.0	6.76	7.60	0.30	20.3	18.0	2.85
Bairinzuoqi1*	43.88	119.36	486	339.0	5.43	8.00	0.35	15.6	22.1	4.96
Bairinzuoqi2*	43.89	119.40	472	353.0	5.26	7.80	0.46	17.0	21.4	4.68
Bairinzuoqi3	43.58	119.23	477	384.0	5.31	7.10	1.18	16.4	20.6	2.54
Baiqi1*	42.24	115.13	1406	363.0	2.06	7.37	1.50	10.7	18.2	6.40
Baiqi2	42.12	115.12	1405	364.0	2.03	7.46	0.48	12.9	18.0	6.37
Tongliao1*	43.61	121.97	207	446.0	5.92	7.93	0.45	19.4	21.5	3.24
Tongliao2	43.63	121.96	202	387.0	6.81	7.60	0.58	16.9	17.9	4.11
Tongliao3	43.60	121.98	205	442.0	5.90	7.83	0.5	17.4	19.4	3.86
